# Social media use motives and academic performances: exploring the mediating role of daily usage duration

**DOI:** 10.3389/fpsyg.2026.1859222

**Published:** 2026-07-09

**Authors:** Ayoob Lone, Saad Habib Alotaibi, Imtiyaz Ahmad Dar, Sayed Abdul Qadar Quadri

**Affiliations:** 1 Department of Clinical Neurosciences, College of Medicine, King Faisal University, Al Ahsa, Saudi Arabia; 2 College of Medicine, King Faisal University, Al Ahsa, Saudi Arabia; 3 Department of Psychology, University of Kashmir, Hazratbal, Srinagar, Jammu and Kashmir, India; 4 Department of Biomedical Sciences, College of Medicine, King Faisal University, Al Ahsa, Saudi Arabia; 5 Department of Medical Education, College of Medicine, King Faisal University, Al Ahsa, Saudi Arabia

**Keywords:** academic performance, medical students, Saudi Arabia, social media motives, social networking sites

## Abstract

**Introduction:**

Research on social networking site (SNS) use and academic performance shows inconsistent results, partly because most studies treat SNS use as a uniform exposure and rarely test motive-specific mechanisms or conditional pathways. Motive-based and mechanism-oriented models remain limited, particularly in medical education and Saudi higher-education contexts.

**Aim and objectives:**

Guided by Uses and Gratifications theory, this study examined a motive-differentiated and mechanism-based model in which SNS use motives relate to academic performance through daily time spent, and examined whether these indirect pathways vary across motivational and student characteristics, and also integrated motive-specific mediation and moderated mediation analyses within a single framework.

**Methods:**

A cross-sectional study was carried out between November, 2025 to February, 2026 involving 323 undergraduate medical students in Eastern Saudi Arabia. SNS motives were assessed using a validated multi-dimensional scale, while daily SNS time and GPA were measured using structured self-report items. Mediation and exploratory moderated mediation analyses were performed using PROCESS with covariate adjustment and 5,000 bootstrap samples.

**Results:**

SNS motives showed selective associations with time spent and GPA. Daily SNS time was negatively associated with GPA across models. Time spent significantly mediated the relationship between socialization motives and GPA, whereas indirect effects for other motives were not robust after adjustment. Informational motives retained a negative direct association with GPA, while academic motives showed a small positive total association. Conditional process analyses further showed that motive → time → GPA pathways varied by co-occurring motives and student characteristics.

**Conclusion:**

By integrating motive differentiation, behavioral mediation, and conditional process modeling in a Saudi medical education setting, this study advances SNS–academic performance research beyond exposure-based approaches and provides novel evidence that academic associations are motive-specific, time-mediated, and context-dependent.

## Introduction

Social media platforms have become deeply embedded in the daily lives of university students on a global scale, shaping communication practices, information access, and learning-related behaviors (Ashraf et al., 2021; Junco, 2012). Over the last decade, social networking sites (SNSs) have transformed from purely social and recreational platforms into multifaceted digital ecosystems that support content creation, interaction, and networking activities (Kaplan and Haenlein, 2010; Kietzmann et al., 2011). In contrast to conventional media, SNSs empower users to actively produce, organize and curate content within participatory online spaces (Boyd and Ellison, 2008). As a result, researchers have increasingly recognized the relevance of SNSs for fostering academic engagement, peer collaboration, and learning practices within higher education contexts (Junco, 2012; Manca and Ranieri, 2016; Tess, 2013). Collectively, these developments underscore the evolution of SNSs from mere social tools into behaviorally relevant learning environments.

Saudi Arabia offers a particularly compelling setting for examining the academic implications of SNS use. The National digital reports estimate that the country has approximately 34.1 million SSN users, representing about 99.6% of the population, with average daily use surpassing 3 h (DataReportal, 2025). Engagement is notably pronounced among young adults aged 18–24, a demographic commonly labeled as Generation Z, who consistently rank among the most active users. University students constitute a substantial segment of this group and demonstrate high levels of platform engagement (Alshanqiti et al., 2023; Alamri, 2019). In light of such pervasive digital engagement, it is pertinent to examine how sustained SNS use may be shaping students’ academic performance, time use patterns, and study behaviors (Alshanqiti et al., 2023; Findyartini et al., 2024).

These concerns are particularly relevant in the context of medical education (Clavier et al., 2024). Medical training is widely acknowledged as cognitively rigorous and performance-oriented, demanding sustained focus, disciplined time management, and advanced self-regulation (Findyartini et al., 2024; Artino, 2012). Meanwhile, SNSs have become integrated into medical students’ learning environments. Students increasingly report using platforms such as YouTube and WhatsApp to share notes, discuss complex course content, and prepare for examinations (Avci et al., 2015; Findyartini et al., 2024). In practice, these platforms often serve as informal academic support networks that extend beyond formal classroom boundaries (Avci et al., 2015; Findyartini et al., 2024). When aligned with clear academic intent, SNS engagement can operate as a meaningful supplementary learning resource rather than a mere distraction (Findyartini et al., 2024; Manca and Ranieri, 2016). This dual potential underscores the need for more theoretically precise investigation into the role of SNSs in medical education.

Despite the exceptionally high rates of SNS penetration in Saudi Arabia and the broader Gulf region, empirical research on this topic remains relatively scarce. A few studies have investigated this issue, such as Alshanqiti et al. (2023) in Medina, Saudi Arabia and Taha et al. (2025) in Jordan, which revealed both positive and negative impacts of social media use on the GPA of medical students. However, these studies have generally conceptualized SNS use as a unitary behavioral exposure, providing limited insight into why students engage with social media and through what mechanisms such engagement influences academic outcomes. Consequently, three important theoretical issues remain unresolved. First, existing research rarely distinguishes among the specific motives that drive SNS engagement, despite evidence that academically oriented, informational, entertainment, and social motives may have different academic implications. Second, little attention has been devoted to the behavioral mechanisms through which these motives translate into academic outcomes. Third, it remains unclear whether such pathways operate uniformly across students or vary according to individual and contextual characteristics. Addressing these gaps may help explain the inconsistent findings reported in previous research and advance a more nuanced understanding of the relationship between SNS use and academic performance.

### Conceptual framework

The inconsistent and often conflicting findings in prior research highlight the need for a theoretical framework capable of explaining why students use social media and how these motives influence academic outcomes. The Uses and Gratifications (U&G) theory, proposed by Katz et al. (1974), offers a suitable analytical lens for this purpose. Unlike traditional media theories that portray audiences as passive consumers of content, U&G emphasizes the active role of users in deliberately selecting media platforms to satisfy specific needs and derive desired gratifications (Ruggiero, 2000). This shift toward an audience-centered perspective underscores that students’ engagement with social networking sites is purposeful, goal-driven, and shaped by underlying psychological and socialization motives.

Within this framework, students engage with SNSs to fulfill a diverse array of needs that encompass cognitive, social, affective, identity-related, and escapist dimensions. Cognitive motives reflect the pursuit of knowledge and academic support, for instance, acquiring information, completing assignments, or preparing for examinations. Socialization motives include the desire to maintain peer relationships, seek emotional or academic support, and expand personal networks. Affective motives relate to the pursuit of entertainment, relaxation, and emotional regulation, whereas personal identity motives reflect self-presentation, self-expression, and the cultivation of social status. Escapist motives, in contrast, represent the use of SNSs for diversion and relief from academic or personal stressors (Quan-Haase and Young, 2010).

Importantly, U&G theory also underscores the dynamic interplay between users’ underlying motives and the extent of daily usage duration, suggesting that daily usage duration may function as a mediating factor influencing academic outcomes. Students’ motives significantly shape the intensity and frequency of their SNS engagement, and this level of involvement subsequently determines whether the academic impact is beneficial or detrimental (Cuong et al., 2025). For instance, cognitively-driven motives may promote purposeful and academically beneficial usage, yet excessive daily use—even for academic purposes—can offset these benefits by displacing focused study time. Likewise, entertainment-driven motives are often associated with prolonged daily use, which may amplify adverse effects on academic performance. Therefore, U&G theory provides a robust theoretical foundation for investigating how students’ motives for SNS use and their daily usage duration together determine the academic consequences of social media engagement.

### Social media use and academic performance

In digital communication research, social media usage is defined as patterns of online behavior that promote interpersonal exchange, such as liking, commenting, sharing and messaging other users (Verduyn et al., 2017). Academic performance, on the other hand, denotes students’ proficiency in fulfilling academic requirements and is evaluated through objective indicators such as GPA and final course results across various subjects (Busalim et al., 2019). In this study, the terms GPA, academic performance, and academic achievement are used interchangeably. The link between social media use motives and university students’ GPA is complex and multidimensional. It is shaped by how students engage with social media, their underlying motives, and the consequent impact on their academic outcomes.

Academic performance is widely regarded as a key determinant of students’ educational attainment, professional development, and future opportunities. However, research examining the relationship between social networking site (SNS) use and academic performance has produced inconsistent findings. Some studies report positive associations, highlighting the educational benefits of SNSs for learning, collaboration, and access to academic resources (Al-Adwan et al., 2020; Alshanqiti et al., 2023; Chao et al., 2025), whereas others document negative associations related to distraction, procrastination, and reduced study efficiency (Taha et al., 2025; Bhandarkar et al., 2021). Still others report no significant relationship (AlFaris et al., 2018; Fauzi et al., 2021; Takieddin et al., 2022). These inconsistencies may partly reflect differences in cultural contexts and variations in how SNS use has been conceptualized and measured across studies. Existing research has operationalized SNS use in diverse ways, including time spent, frequency of use, and problematic use, often treating SNS engagement as a single behavioral construct despite the heterogeneous nature of social media platforms and user motivations (Aichner et al., 2021). Consequently, examining the motives underlying SNS engagement may provide a more theoretically informative explanation for the mixed findings reported in the literature.

Consistent with Uses and Gratifications theory, the present study therefore focuses on SNS use motives rather than usage behavior alone, as different motives may have distinct implications for academic performance.

### Academic motives and academic performance

The academic value of SNS’s is closely tied to the purposes for which students engage with them. When SNS’s are used with explicit academic motives, they can facilitate collaboration, promote information sharing, and provide access to valuable study materials, thereby supporting improved academic performance. For instance, Le et al. (2023) reported that the use of SNSs as learning collaboration tools improved students’ ability to transfer knowledge effectively and improved group learning outcomes. In support of these findings, Junco et al. (2011) experimental results demonstrated that students who made use of Twitter in their learning activities had significantly higher levels of class participation and achieved better grades compared with those who did not use the platform for academic purposes. Collectively, these findings indicate that SNSs can serve as effective learning tools if accessed with purposeful, academically oriented motivations.

Systematic reviews and large-scale surveys similarly suggest that academically oriented SNS use is associated with improved learning outcomes and higher academic achievement (Gao, 2021; Hughes-Nind et al., 2024).

*H1:* Academic motives for social media use are positively associated with students’ GPA.

### Entertainment motives and academic performance

Social networking site use for entertainment purposes has been widely regarded as a factor that hinders academic performance (Cuong et al., 2025). Unlike academic motives, which promote collaboration and learning, entertainment motives often lead to distraction (Feng et al., 2019; Gao, 2021), procrastination, and reduced study efficiency. Students who make SNSs the primary source of enjoyment, i.e., watching videos, following celebrity preferences, or uploading gaming-related posts, are also likely to waste significant amounts of time on non-academic activities. This diversion of time, attention and energy can interfere with attention to studies and exam preparation, thereby affecting GPA in a negative manner (Cuong et al., 2025; Kalam et al., 2023; Masalimova et al., 2023). Previous research has consistently demonstrated that when social media is employed predominantly for entertainment purposes, it is often associated with lower academic achievement (Zavala et al., 2023; Feng et al., 2019; Gao, 2021). Such outcomes are commonly attributed to reduced self-regulation and ineffective time management among students who prioritize leisure-oriented online engagement.

Entertainment-oriented SNS use may also disrupt concentration and reduce study efficiency by encouraging frequent interruptions during academic activities (Wood et al., 2012; Hu and Yu, 2021). Based on the above theoretical and empirical discussion, the following hypothesis is formulated.

*H2:* Entertainment motives for social media use are negatively associated with students’ GPA.

### Information motives and academic performances

Informational motives refer to the intrinsic drives to seek, obtain, and apply knowledge in order to accomplish specific objectives. Within the context of social networking sites, students often use these platforms not only for entertainment or social interaction but also for accessing educational content, staying informed about relevant academic issues, communicating with peers or mentors who can aid their learning, and exploring career-related opportunities (Gupta and Bashir, 2018). Compared with conventional sources of information, SNSs provide immediacy, interactivity, and personalization in information content, allowing learners to explore, locate, organize, and utilize information according to their individual needs.

From a learning perspective, information-oriented use of SNSs use may support academic performance by providing rapid access to educational resources, facilitating knowledge exchange, and exposing students to diverse perspectives that can enhance learning and critical thinking. Through these mechanisms, SNSs may function as supplementary sources of information that support academic engagement and knowledge acquisition (Gupta and Bashir, 2018; Ibrahim et al., 2024).

Despite the possible benefits, empirical research on the relationship between information-oriented SNS usage and academic performance remains limited (Cuong et al., 2025). While some studies highlight its role in enhancing learning and academic achievement (Ibrahim et al., 2024), others have proposed that the overwhelming volume of information available on SNSs may contribute to information overload and difficulties in information processing. Such mechanisms have been suggested as potential explanations for poorer academic outcomes, although empirical findings remain mixed (Ather et al., 2024). Consequently, further research is needed to better understand how informational motives influence academic performance and how potential drawbacks can be mitigated. Evidence also suggests that the effects of information-oriented SNS use may vary across demographic and cultural contexts. For example, Zavala et al. (2023) reported that, among African American students, information use via SNSs was correlated with lower GPA. In contrast, this relationship was not observed among Vietnamese students (Cuong et al., 2025). However, information motives even showed a positive correlation with GPA in Hispanic students (Zavala et al., 2023). Existing research, though limited, suggests that students’ information management abilities and demographic features may influence whether informational SNS use benefits or hinders academic performance. Therefore, the following hypothesis is propounded.

*H3:* Information motives in social networking site usage are positively associated with students’ GPA.

### Socialization motives and academic performances

Socialization motives are one of the most common reasons that students utilize social networking websites. Socialization motives include the desire to communicate with peers, establish and maintain friendships, share experiences, and receive social support (Gebremariam et al., 2024). For many students, social media platforms function as virtual communities that not only expand interpersonal relationships but also provide a space for academic collaboration and group discussions (Junco, 2012).

Socialization through SNSs may improve academic performance if interactions center on the exchange of learning materials, task discussions, or study groups. Research has shown that collaborative learning and peer support through social media can enhance student engagement and improve learning outcomes (Manca and Ranieri, 2016). Similarly, Chen and Bryer (2012) noted that social media facilitates a participatory culture in which students share learning and collaboratively construct knowledge, potentially leading to improved academic achievement.

However, when socialization motivation is primarily entertainment-oriented— such as casual chatting, posting personal updates, or engaging in non-academic arguments— detrimental consequences will ensue. Studies have established that excessive social media use solely for social purposes can lead to distraction, wasting time, and procrastination, all of which are associated with poor academic performance (Yeboah and Ewur, 2014). For example, Junco (2012) identified an inverse relationship between the time spent on social media and GPA among the students, particularly when the use is unrelated to academics.

Accordingly, the academic implications of socialization motives depend largely on whether SNS interactions are oriented toward learning-related collaboration or non-academic engagement (Al-Menayes, 2015).

*H4:* Socialization motives for using social networking sites are significantly associated with academic performance among students.

### Time allocation as a behavioral mechanism

U&G theory further posits that motives influence not only the type of engagement but also its intensity (Quan-Haase and Young, 2010). Daily SNS usage duration, therefore, represents a behavioral allocation mechanism through which motivational drivers may influence academic outcomes (Al-Menayes, 2015). A growing body of evidence links extended daily SNS use with lower academic performance, often attributing this association to attentional fragmentation and time displacement (Leyrer-Jackson and Wilson, 2018; Junco, 2012; Karpinski et al., 2013). Recent mediation analyses suggest that time spent on SNSs may serve as an explanatory pathway connecting SNS motives to academic outcomes such asGPA (Cuong et al., 2025). Given the high SNS penetration in Saudi Arabia (DataReportal, 2025; Alamri, 2019) and the intensive cognitive demands associated with medical education (Findyartini et al., 2024), examining time allocation as a mediating mechanism is theoretically and contextually warranted. Therefore, the following hypothesis is formulated.

*H5:* Daily time spent on SNSs is negatively associated with GPA.

*H6:* Daily time spent mediates the relationship between SNS use motives and GPA.

### Conceptual integration and conditional extensions

In line with Uses and Gratifications (U&G) theory, SNS use motives in this study are categorized into academic, informational, socialization, and entertainment dimensions, each reflecting distinct patterns of engagement. Among these, particular attention is given to distinguishing academic and informational motives, as both involve knowledge-related use but differ in important ways. Academic motives refer to structured, goal-directed engagement directly linked to coursework, such as preparing for examinations or completing assignments. In contrast, informational motives reflect broader and less structured knowledge-seeking behaviors that may not be tied to immediate academic tasks. Thus, the distinction lies primarily in goal specificity and alignment with formal academic requirements. This distinction is further reflected in the content of the items, which vary in their level of goal specificity and enable respondents to differentiate between coursework-oriented and more general information-seeking use. This conceptual distinction is consistent with the original multidimensional structure proposed by Gupta and Bashir (2018) and with recent applications of the instrument that have interpreted these dimensions as distinct SNS use motives (Cuong et al., 2025).

**FIGURE 1:**
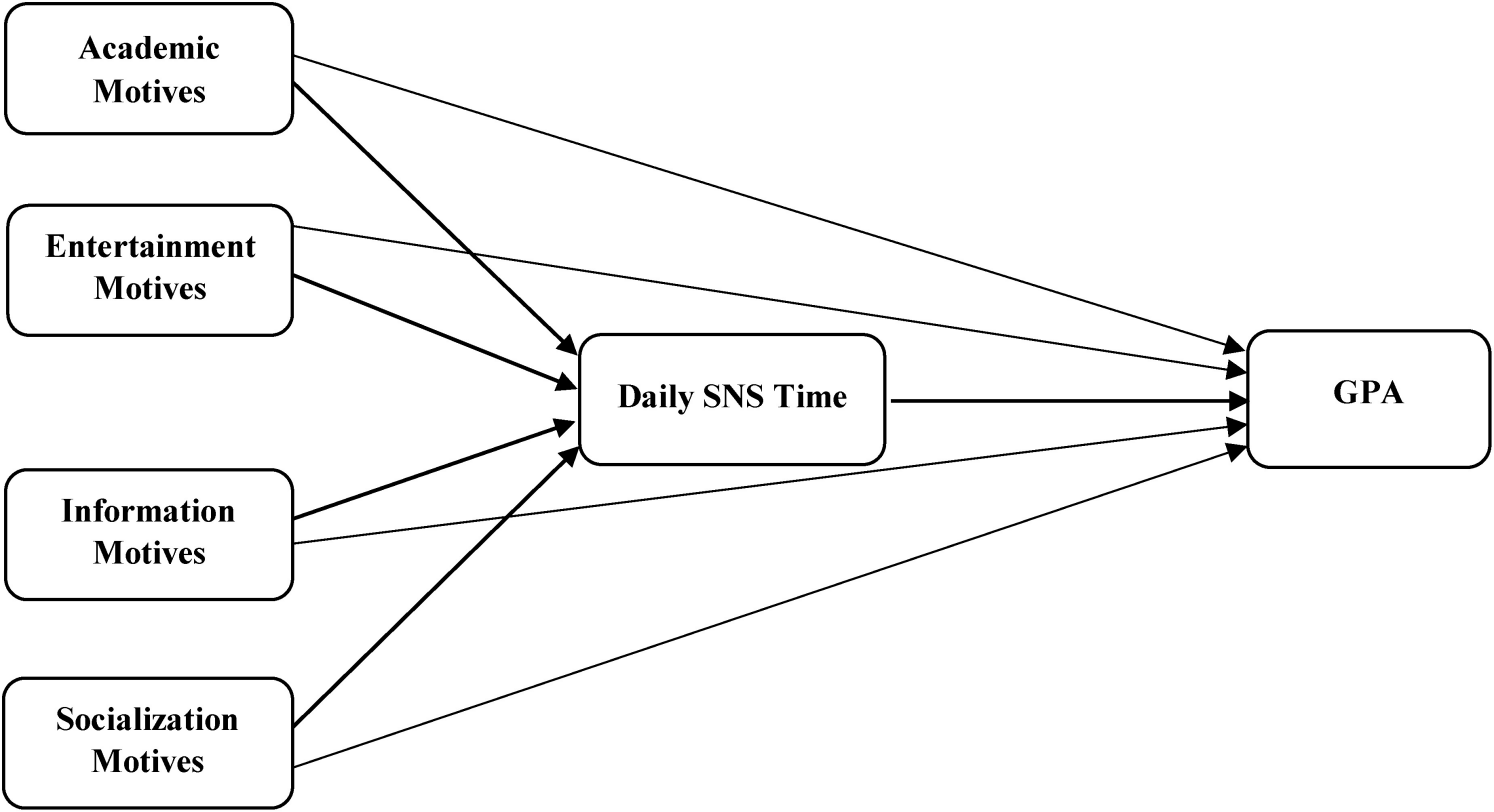
Conceptual mediation model linking social networking site use motives (academic, entertainment, informational, and socialization) with academic performance (GPA) through daily SNS time. Direct and indirect pathways were estimated, with exploratory conditional effects examined on the motive–time pathway.

Grounded in U&G theory (Katz et al., 1974; Ruggiero, 2000), the present study advances a motive-differentiated mediation model in which academic, informational, entertainment, and socialization motives function as parallel predictors of GPA, with daily SNS time specified as a behavioral exposure pathway linking motivational drivers to academic outcomes. By integrating motive differentiation, time allocation mechanisms, and exploratory conditional process analyses within a single framework, the study extends beyond exposure-based approaches and clarifies how and under what conditions SNS engagement translates into academic performance. Conditional effects are examined as theoretically informed extensions rather than confirmatory subgroup hypotheses. Figure 1 presents the proposed mediation model.

## Materials and methods

### Study design

An institution-based cross-sectional study was carried out in November, 2025 and February 2026 at King Faisal University, located in the Eastern governorate of Saudi Arabia. Using the convenient sampling approach, participants were recruited from undergraduate students enrolled in the University. The study protocol received ethical approval from the Deanship of Scientific Research at King Faisal University, Saudi Arabia (KFU-REC-2025-AUG–ETHICS3463). All procedures were conducted in accordance with the Declaration of Helsinki and other relevant ethical guidance for research involving human participants (World Medical and Association, 2013). Before participation, students were provided with detailed information regarding the study objectives, procedure, and confidentiality of their responses. Participation was entirely voluntary, and informed consent was obtained from all participants. Data was collected only after ensuring that all ethical requirements were fully satisfied.

### Study sample

The target population consisted of undergraduate medical students enrolled at the College of Medicine, King Faisal University. At the time of data collection, the total enrolled student population was 1,639. The minimum required sample size was calculated using Yamane’s (1967) formula for finite populations with a 5% margin of error (Adam, 2020), resulting in a target of 322 participants. A total of 400 students were approached to take part in the study. Of these, 339 agreed to participate (agreement rate = 84.8%). Sixteen returned questionnaires contained substantial missing information and were excluded prior to analysis. Following standard data screening procedures, including assessment of multivariate outliers (described in the Statistical analysis section), one additional case was removed. The final analytic sample, therefore, comprised 323 students, representing approximately 80.8% of those initially approached. The final sample included 165 male students (51.1%) and 158 female students (48.9%), with ages ranging from 19 to 25 years (*M* = 21.6, SD = 1.7). Students from all five academic years were represented: 19.8% were in year 1, 27.6% in year 2, 18.0% in year 3, 21.7% in year 4, and 13.0% in year 5. Regarding monthly household income, 10.8% reported < 5,000 SAR, 24.5% reported 5,001–10,000 SAR, 20.1% reported 10,001–15,000 SAR, and 44.6% reported more than 15,001 SAR.

To ensure adequate statistical sensitivity, sample size adequacy was evaluated prior to analysis. An initial estimate was obtained using G*Power (Faul et al., 2009). Assuming a conventional alpha level of 0.05, statistical power of 0.80, and a small effect size (*f*^2^ = 0.05; Cohen, 1988), the calculation suggested that roughly 235 participants would be adequate for regression models including up to eight predictors. Because the primary focus of the study was the indirect pathway rather than a single direct association, power for the mediation effect was examined separately using a Monte Carlo simulation approach (Schoemann et al., 2017). Parameter values were selected to reflect effect sizes commonly observed in prior work linking social media use and academic performance (Huang, 2018; Cuong et al., 2025). Specifically, standardized estimates of *a* = 0.25 and *b* = –0.17 were specified, along with a modest direct effect (*c*’ = –0.10), corresponding to a small indirect effect overall. Across 20,000 replications at α = 0.05, the simulation indicated that a sample of approximately 300 participants would yield 80% power. The final analytic sample of 323 students, therefore, provided adequate sensitivity for detecting effects of this magnitude.

### Data collection tools

In order to achieve the goal of this study, we used a structured questionnaire consisting of two parts. The first part of the survey collected data on different socio-demographic information, specifically sex, age, academic years, number of social networking sites accounts and family income. Additionally, participants were asked to provide their GPA for the previous semester, measured on a scale out of 5, with 1 representing a GPA between 4.5 and 5.0, and 5 representing a GPA below 2.0. This 5-point scale allowed for a standardized assessment of academic performance across participants. Importantly, GPA was measured using a five-point categorical scale, with higher values initially indicating lower academic performance; this was reverse-coded so that higher scores reflected better performance in the analyses. Instead of asking students to report their exact GPA, we used predefined ranges, as students may not always recall precise scores or may feel uncomfortable reporting them. Using categories also helped reduce response burden and ensured that most participants were able to provide this information. For the analyses, each category was represented by its midpoint (for example, 4.75 for the 4.5–5.0 range), which allowed GPA to be treated as approximately continuous in the regression models. This step made the analyses more straightforward, although it should be noted that it does not capture the full precision of actual GPA values. To check that our findings were not dependent on this decision, we also ran ordinal regression models using GPA in its original categorical form, and the overall pattern of results remained similar. Moreover, we measured total screen time spent on social media, we adapted an item from previous researches (Cuong et al., 2025). Participants were asked to report the total time they spend on social media each day. Responses were recorded using a 7-point scale where “1” indicated 1 h or less and “7” represented 7 h or more of daily social media use. In the second section of the questionnaire, we included the Social Networking Usage Questionnaire (SNUQ) developed by Gupta and Bashir (2018). This 19-item scale comprised of four aspects of the use of social media, such as academic (7 items), socialization (5 items), entertainment (4 items), and informativeness (3 items). A 5-point Likert scale with “1” representing “never” and “5” representing “always” was used to assess respondent’s use of social networking sites. In this measure, a higher score indicates higher usage of social networks. Although the SNUQ was not originally developed as a motive scale, it possesses the necessary dimensions of students’ motives for using social networking sites, which have been determined in recent empirical studies (AlFaris et al., 2018; Masalimova et al., 2023). Most importantly, the instrument was originally validated in the Indian context and has been widely used in Arab settings (Ibrahim et al., 2024; Hama and Mohammad, 2024). Although the SNUQ was not originally developed as a motive-based instrument, its four subdimensions correspond closely to commonly examined Uses and Gratifications (U&G) domains, including academic, socialization, entertainment, and informational gratifications. Consequently, in the present study these dimensions were interpreted as indicators of gratification-oriented SNS engagement rather than direct psychometric measures of latent motives. This interpretation is consistent with recent applications of the instrument. For example, Cuong et al. (2025) employed the same four dimensions as SNS use motives and reported support for the corresponding four-factor structure through confirmatory factor analysis. Accordingly, the present study adopts a similar conceptual interpretation while acknowledging that the SNUQ was originally developed to assess patterns of SNS use. In the original validation study, the SNUQ demonstrated good internal consistency, with a Cronbach’s alpha of 0.83, supporting its reliability. In the original validation study, the scale demonstrated good internal consistency (Cronbach’s α = 0.83). Reliability estimates for the present sample are presented in Table 2.

### Procedure

Following institutional approval, student representatives from each academic cohort were contacted to assist with recruitment. Each cohort maintained a WhatsApp group primarily used for academic communication, and with permission from group administrators, information about the study was shared with group members. Recruitment through WhatsApp-based academic groups may have introduced selection bias, volunteer bias, and an overrepresentation of students who are more digitally active and frequently engaged with social networking platforms. Consequently, students with lower levels of digital engagement or limited participation in online academic communication may have been less likely to be reached or to participate. This recruitment strategy may therefore have influenced the distribution of social media use within the sample and should be considered when interpreting the findings and their generalizability. Students who expressed interest were subsequently approached in person by trained senior medical students responsible for data collection. Prior to completing the questionnaire, participants received a brief explanation of the study’s purpose and procedures. Written informed consent was obtained, and students were reminded that participation was voluntary and that their responses would remain confidential. The questionnaire was completed individually using a paper-based format and required approximately 10–15 min to complete. Upon submission, participants were thanked for their time and contribution.

### Statistical analysis

Before turning to the primary analyses, the dataset was reviewed in detail to confirm that it was suitable for statistical testing and free from entry-related errors. All survey responses were coded and entered into Statistical Package for the Social Science (SPSS, IBM, Version 27), after which the data file was examined line by line to identify any inconsistencies or miscoding. This process did not reveal any problems. In addition, all participants completed the survey in full, and as a result, no missing data were present. The data were then screened for outliers. Univariate outliers were assessed using standardized z-scores, with values exceeding ± 3.29 treated as extreme at the *p* < 0.001 level (Field, 2013; Tabachnick and Fidell, 2013). None of the cases met this criterion. Multivariate outliers were examined separately using Mahalanobis distance. Applying the chi-square cutoff for nine predictors at *p* < 0.001, one case exceeded the threshold and was removed prior to further analyses (Field, 2013). No additional multivariate outliers were identified.

Normality was evaluated by inspecting skewness and kurtosis values for all study variables. Following commonly cited guidelines (Hair et al., 2010; Kline, 2016; Tabachnick and Fidell, 2013), skewness values within ± 3 and kurtosis values within ± 10 were considered acceptable for multivariate procedures. In the present dataset, skewness values ranged from –1.34 to 0.19, while kurtosis values ranged from –1.26 to 2.46. Taken together, these values indicate that the variables were approximately normally distributed. Multicollinearity was also assessed to ensure that the predictor variables were not excessively overlapping. Tolerance and Variance Inflation Factor (VIF) values were examined in line with standard recommendations (Field, 2013; Hair et al., 2010; Kline, 2005). Although most predictors fell within acceptable limits, age and academic year showed notably elevated VIF values, suggesting substantial shared variance (Table 1). Because the academic year was considered more meaningful for interpreting students’ academic progression. Age was removed from the regression models. After its removal, all remaining predictors showed acceptable tolerance and VIF values, indicating that multicollinearity was no longer problematic. Finally, common method bias was examined using Harman’s one-factor test. As noted by Podsakoff et al. (2003), common method bias may be a concern if a single factor accounts for more than half of the total variance. In this study, the first unrotated factor accounted for 27.33% of the variance, suggesting that common method bias was unlikely to pose a serious threat to the validity of the findings. However, Harman’s single-factor test is a limited diagnostic tool and cannot definitively rule out the presence of common method variance. To mitigate potential bias at the design stage, several procedural remedies were implemented. Participants were assured of full anonymity and confidentiality, which reduces evaluation apprehension and socially desirable responding. In addition, the questionnaire employed previously validated instruments with conceptually distinct subscales representing different constructs (e.g., motives, time use, and academic performance), thereby reducing item ambiguity and construct overlap. Furthermore, the predictor, mediator, and outcome variables were measured using different item formats and scale structures, which helps reduce method-related inflation of associations. Because the Social Networking Usage Questionnaire (SNUQ) was originally developed to assess patterns of SNS use rather than motives *per se*, additional analyses were conducted to evaluate the distinctiveness of the study constructs within the present sample. Discriminant validity was assessed using the Fornell–Larcker criterion and the heterotrait–monotrait ratio of correlations (HTMT). Following established recommendations, discriminant validity was considered satisfactory when the square root of the average variance extracted (AVE) for each construct exceeded its correlations with other constructs and when HTMT values were below 0.85 (Fornell and Larcker, 1981; Henseler et al., 2015). Hypotheses were tested using Hayes’ PROCESS macro (Version 4; Hayes, 2018). Simple mediation analyses were conducted using Model 4, whereas moderated mediation models were estimated using Model 7, specifying the moderation of the path from the predictor to the mediator. These moderated mediation analyses were specified as exploratory and hypothesis-generating rather than confirmatory, given the absence of strong a priori hypotheses regarding specific moderation effects. Indirect and conditional indirect effects were examined using 5,000 bootstrap resamples to generate bias-corrected 95% confidence intervals. Effects were considered statistically significant when the corresponding confidence interval did not include zero. This approach is recommended for mediation analysis because it improves statistical power and provides more accurate control of Type I error compared to normal-theory tests (Fritz et al., 2016;Hayes, 2018).

**TABLE 1:** Multicollinearity statistics and bivariate correlations among study variables (*N* = 323).

Variable	1	2	3	4	5	6	7	8	9	10	Tolerance	VIF
1. GPA	–	–	–	–	–	–	–	–	–	–	–	–
2. Time spent on SNS	–0.21**										0.92	1.09
3. Socialization motives	–0.08	0.18**									0.56	1.79
4. Academic motives	0.05	0.05	0.50**								0.62	1.60
5. Information motives	–0.12*	0.12*	0.59**	0.47**							0.60	1.67
6. Entertainment motives	–0.06	0.14*	0.26**	0.36**	0.20**						0.81	1.23
7. Age	–0.06	0.05	0.11	0.11*	0.16**	0.06					0.10	9.71
8. Academic year	–0.02	0.07	0.13*	0.18**	0.18**	0.08	0.94**				0.10	9.63
9. Family income	0.18**	0.00	–0.11*	–0.02	–0.08	0.04	–0.13*	–0.08			0.82	1.22
10. Number of SNS accounts	0.11*	0.16**	–0.03	0.04	–0.00	0.21**	–0.21**	–0.14**	0.40**		0.74	1.36

*p < 0.05, **p < 0.01.

**TABLE 2:** Descriptive statistics and reliability indices for the social networking usage questionnaire (*N* = 323).

Factor/item	Mean	SD	Skewness	Kurtosis	Cronbach’s alpha	Item–total correlation	Cronbach’s alpha if item deleted
Academic motives					0.809	–	–
AI	2.755	1.221	0.261	–0.932			
A2	3.619	1.224	–0.468	–0.831		0.533	0.786
A3	3.415	1.307	–0.291	–1.052		0.586	0.777
A4	3.489	1.217	–0.422	–0.685		0.568	0.780
A5	3.848	1.139	–0.588	–0.713		0.538	0.786
A6	3.499	1.199	–0.383	–0.727		0.617	0.772
A7	3.511	1.209	–0.455	–0.650		0.532	0.787
Entertainment motives					0.783	–	–
E1	3.864	0.867	–0.366	–0.410			
E2	3.873	1.114	–0.574	–0.743		0.558	0.746
E3	3.588	1.271	–0.459	–0.925		0.505	0.787
E4	4.099	1.053	–1.035	0.280		0.457	0.793
Information motives					0.737	–	–
I1	3.102	1.378	–0.049	–1.235			
I2	3.006	1.316	0.079	–1.140		0.474	0.761
I3	2.808	0.969	0.145	–0.299		0.744	0.507
Socialization motives					0.820		
SI	3.226	1.214	–0.116	–0.917			
S2	3.028	1.090	0.162	–0.552		0.927	0.701
S3	3.028	1.267	–0.071	–0.945		0.658	0.772
S4	2.910	1.404	0.045	–1.260		0.605	0.790
S5	3.520	1.217	–0.380	–0.852		0.378	0.849

## Results

### Bivariate correlations

Bivariate correlations among all study variables are presented in Table 1. Several demographic variables, including age, academic year, family income, and number of social media accounts, showed significant associations with the independent, dependent, and mediating variables. These patterns indicate that demographic characteristics were related to key study constructs at the zero-order level. Because these variables showed systematic relationships with the main constructs, such as academic year, family income, and number of social network accounts were retained as control variables in subsequent models so that the focal effects could be estimated under adjusted conditions. Sex was included as a control variable due to documented differences in social media use and academic impact between male and female medical students (Alnjadat et al., 2019). Additionally, although academic and informational motives were moderately correlated, the magnitude of the association remained within acceptable limits, indicating that the constructs are related but empirically distinct.

### Psychometric properties of the measurement scales

As presented in Table 2, the scale demonstrated strong performance within the present sample. Item mean scores ranged from 2.81 to 4.10, reflecting appropriate endorsement levels and sufficient variability across items. Skewness values (–1.04 to 0.26) and kurtosis values (–1.26 to 0.28) fell within acceptable thresholds, indicating no substantial deviations from normality. The four subscales exhibited acceptable to good internal consistency, with Cronbach’s alpha coefficients ranging from 0.74 to 0.82. Item–total correlations were generally moderate to high (0.38—0.95), and the removal of any item did not result in notable improvements in reliability. Taken together, these findings support the reliability and psychometric adequacy of the scales for this study sample.

### Discriminant validity

Discriminant validity was evaluated using the Fornell–Larcker criterion and the heterotrait–monotrait ratio of correlations (HTMT). Results indicated adequate discriminant validity across all study constructs. Specifically, the square root of the average variance extracted (AVE) for each construct exceeded its correlations with all other constructs, satisfying the Fornell–Larcker criterion. Likewise, all HTMT values were below the recommended threshold of 0.85, ranging from 0.247 to 0.740. These findings indicate that the four SNS motive dimensions represent empirically distinct constructs.

Given the conceptual overlap between Academic Motives and Informational Motives noted in the literature, additional attention was paid to this construct pair. The square roots of the AVEs for Academic Motives (0.618) and Informational Motives (0.770) both exceeded their inter-construct correlation (*r* = 0.555), and the corresponding HTMT value (0.599) remained well below the recommended threshold. These results provide empirical support for treating Academic Motives and Informational Motives as separate constructs in the subsequent regression, mediation, and moderated mediation analyses.

### Mediation analyses

Mediation analyses were conducted using PROCESS Model 4 in SPSS (Version 3.5; Hayes, 2018) with 5,000 bias-corrected bootstrap resamples to examine whether time spent on social networking sites accounted for the relationship between social media motives and GPA. Each mediation model was initially estimated without covariates and subsequently re-estimated with demographic and usage controls to assess the robustness of the indirect effects (Hayes, 2018). Regression coefficients are reported in Table 3, while bootstrapped indirect effects are presented in Table 4.

**TABLE 3:** Mediation models predicting GPA through time spent on social networking sites (PROCESS model 4; *N* = 323).

Predictor	Without covariates				With covariates			
	B	β	SE	*t*	B	β	SE	*t*
Mediator model (X → M)								
Academic motives	0.014	0.047	0.017	0.850	–0.038	–0.127	0.021	–1.787
Entertainment motives	0.069*	0.135*	0.029	2.433	0.042	0.082	0.031	1.368
Informational motives	0.072*	0.124*	0.032	2.233	0.020	0.034	0.041	0.492
Socialization motives	0.064**	0.176**	0.020	3.195	0.060*	0.163*	0.027	2.178
Outcome model (M → Y)								
Time spent	–0.068***	–0.206***	0.018	–3.710	–0.070***	–0.215***	0.018	-3.871
Total effects (c)								
Academic motives → GPA	0.005	0.052	0.006	0.937	0.015*	0.148*	0.007	2.079
Entertainment motives → GPA	–0.010	–0.061	0.009	–1.087	–0.016	–0.097	0.010	–1.602
Informational motives → GPA	–0.023*	–0.119*	0.011	–2.154	–0.029*	–0.154*	0.013	–2.193
Socialization motives → GPA	–0.010	–0.079	0.007	–1.414	–0.007	–0.057	0.009	–0.757
Direct effects (c’)								
Academic motives → GPA	0.006	0.063	0.005	1.145	0.012	0.121	0.007	1.725
Entertainment motives → GPA	–0.006	–0.033	0.009	–0.589	–0.013	–0.079	0.010	–1.334
Informational motives → GPA	–0.018	–0.094	0.010	–1.724	–0.028*	–0.147*	0.013	–2.133
Socialization motives → GPA	–0.005	–0.043	0.007	–0.768	–0.003	–0.022	0.009	–0.295

* *p* < 0.05, ** *p* < 0.01, *** *p* < 0.001. B, unstandardized coefficient; β, standardized coefficient. Covariates in the adjusted model included remaining social media use motives, sex, academic year, family income, and number of social networking sites accounts.

**TABLE 4:** Indirect effects of social media motives on GPA via time spent on social networking sites (PROCESS model 4; *N* = 323).

Indirect path (a × b)	Without covariates				With covariates			
	B	Boot SE	95% Bootstrap CI	β (cs)	B	Boot SE	95% Bootstrap CI	β (cs)
Academic motives → time → GPA	–0.001	0.001	[–0.0033, 0.0011]	–0.01	0.003	0.002	[–0.0003, 0.0068]	0.03
Entertainment motives → time → GPA	–0.005	0.002	[–0.0096, –0.0008]	–0.03	–0.003	0.002	[–0.0076, 0.0012]	–0.02
Informational motives → time → GPA	–0.005	0.003	[–0.0101, –0.0006]	–0.03	–0.001	0.003	[–0.0077, 0.0041]	–0.01
Socialization motives → time → GPA	–0.004	0.002	[–0.0085, –0.0013]	–0.04	–0.004	0.002	[–0.0095, –0.0004]	–0.04

*B*, unstandardized indirect effect; Boot *SE*, bootstrap standard error; β(cs), completely standardized indirect effect, interpreted as an indicator of the magnitude of the mediation pathway. Confidence intervals are bias-corrected bootstrap intervals based on 5,000 samples. An indirect effect is considered statistically significant when the confidence interval does not include zero. In the covariate-adjusted model, remaining social media use motives, sex, academic year, family income, and number of social networking sites accounts were included as controls.

The overall regression equations were statistically significant. The mediator model predicting time spent on social networking sites was significant, *F*(8, 314) = 3.39, *p* = 0.001, explaining 7.96% of the variance (*R*^2^ = 0.080). The full outcome model predicting GPA was also significant *F*(9, 313) = 4.52, *p* < 0.001, accounting for 11.49% of the variance (*R*^2^ = 0.115). The corresponding total-effect models (excluding the mediator) were likewise significant, *F*(8, 314) = 3.07, *p* = 0.002, explaining 7.25% of the variance in GPA (*R*^2^ = 0.073). Although several associations were statistically significant, the effect sizes were generally small to modest, and the models explained a limited proportion of variance in GPA, indicating modest practical impact.

Across models, time spent on social networking sites showed a consistent negative association with GPA. In the unadjusted model, this association was statistically significant (β = –0.21, *p* < 0.001), and it remained significant after covariate adjustment, indicating that the mediator–outcome relationship was robust.

Regarding indirect effects, bootstrap analyses showed that socialization motives had a statistically significant indirect effect on GPA through time spent, both before adjustment [β(cs) = –0.04, 95% CI (–0.0085, –0.0013)] and after adjustment [β(cs) = –0.04, 95% CI (–0.0095, –0.0004)]. In contrast, academic motives did not exhibit a significant indirect effect in either model. Informational and entertainment motives showed significant indirect effects in unadjusted models, but these estimates were attenuated and no longer statistically reliable once covariates were included (Table 4). Completely standardized indirect effects (βcs), together with their bootstrapped confidence intervals, are reported to aid interpretation of the magnitude and reliability of the mediation effects.

Total and direct effects of social media motives on GPA are presented in Table 4. At the total effect level, academic motives were modestly positively associated with GPA (β = 0.148, *p* < 0.05), whereas informational motives were negatively associated (β = –0.154, *p* < 0.05), and entertainment and socialization motives showed no significant total effects. At the direct effect level, none of the motives demonstrated significant associations in the unadjusted models. After including daily time spent on social networking sites and adjusting for covariates, only informational motives retained a significant direct negative effect (β = –0.147, *p* < 0.05), while academic, entertainment, and socialization motives no longer had direct effects.

Taken together, the mediation results indicate that time spent on social networking sites operates as a statistical intermediary pathway primarily for socialization motives, for which a significant indirect effect was observed after covariate adjustment. In contrast, indirect effects for other motives were attenuated and did not reach statistical significance, and several predictor-to-mediator (X → M) paths were reduced after adjustment. Informational motives, however, retained a direct association with GPA. This pattern raises the possibility that the magnitude of the first-stage pathway may vary across individual characteristics rather than remaining uniform across the sample, warranting further examination through moderated mediation analyses. Accordingly, conditional process models were estimated to examine whether the strength of the indirect pathway differed across levels of the proposed moderators, with moderation specified on the X → M pathway (Hayes, 2018).

### Moderated mediation analyses

Moderated mediation analyses were conducted using PROCESS Model 7 to test whether the indirect effects of social media use motives on GPA through time spent on social networking sites varied across individual and contextual characteristics. In all models, remaining motives and demographic variables were included as covariates. Academic year, family income, and number of social media accounts were treated as multi-categorical moderators and dummy-coded with the lowest category as the reference group.

**FIGURE 2:**
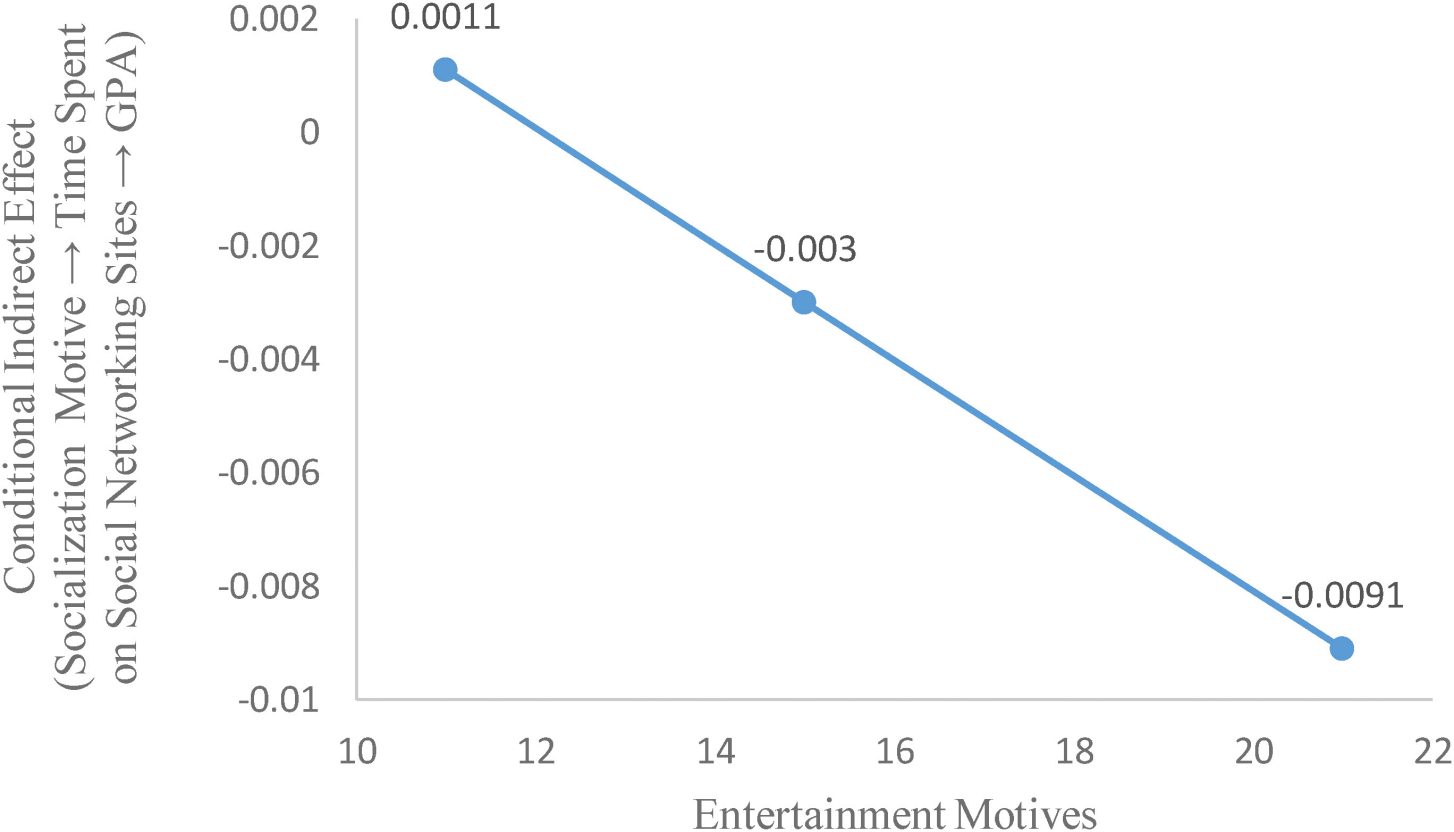
Conditional indirect effect of socialization motives on GPA through time spent on social networking sites across levels of entertainment motives.

**FIGURE 3:**
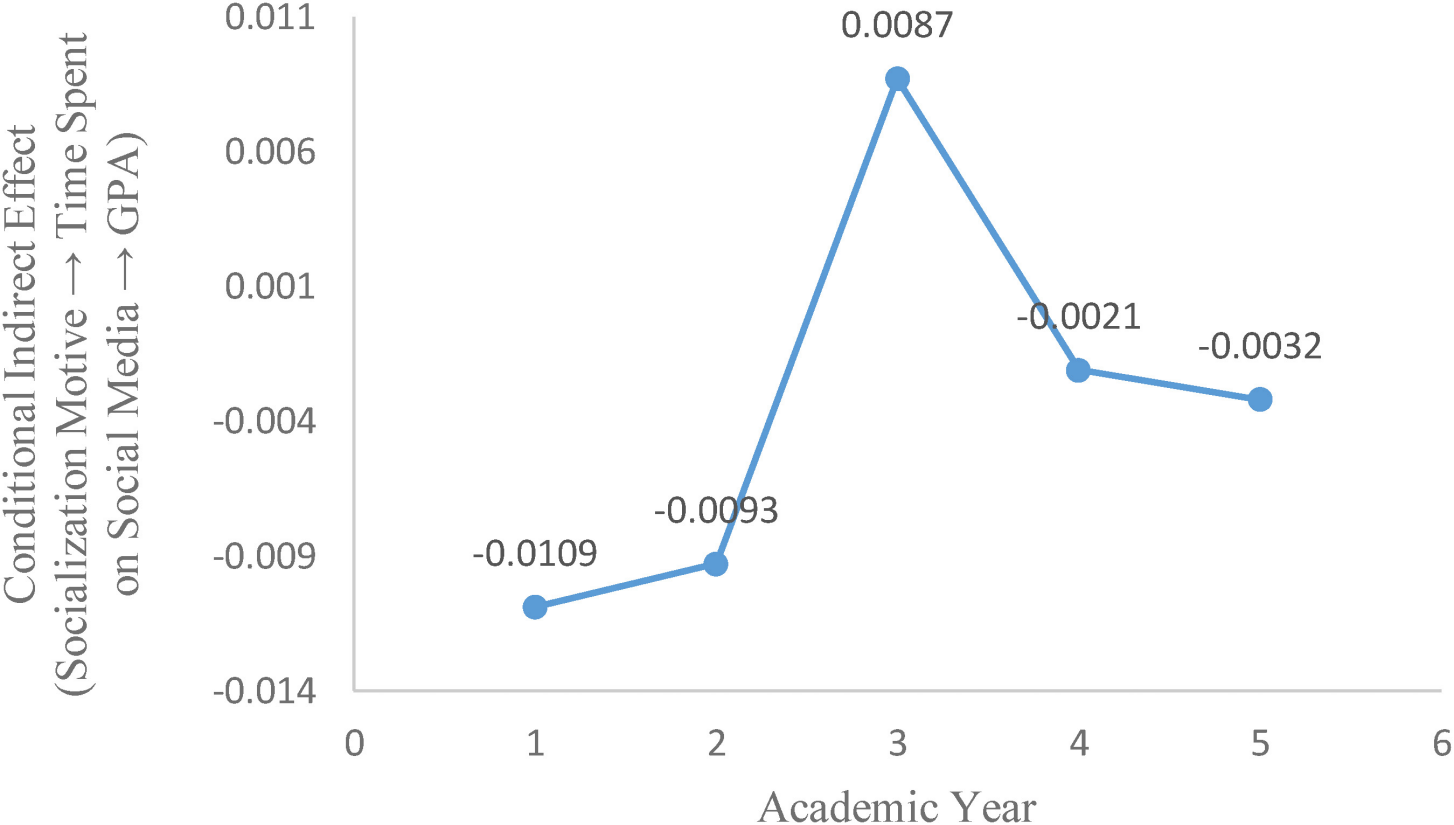
Conditional indirect effect of socialization motives on GPA through time spent on social networking sites across levels of academic year.

**FIGURE 4:**
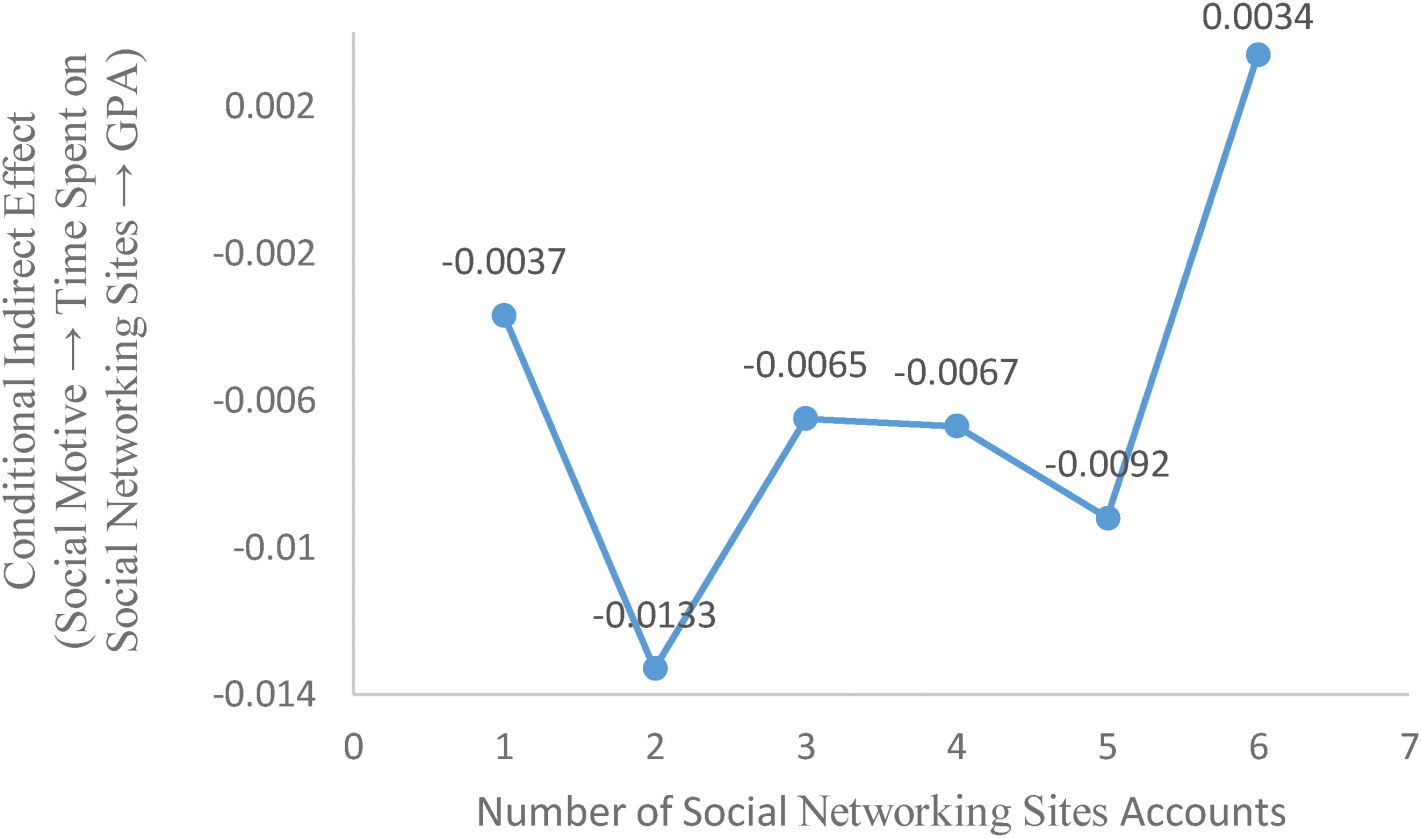
Conditional indirect effect of socialization motives on GPA through time spent on social networking sites across number of social networking sites accounts categories.

**FIGURE 5:**
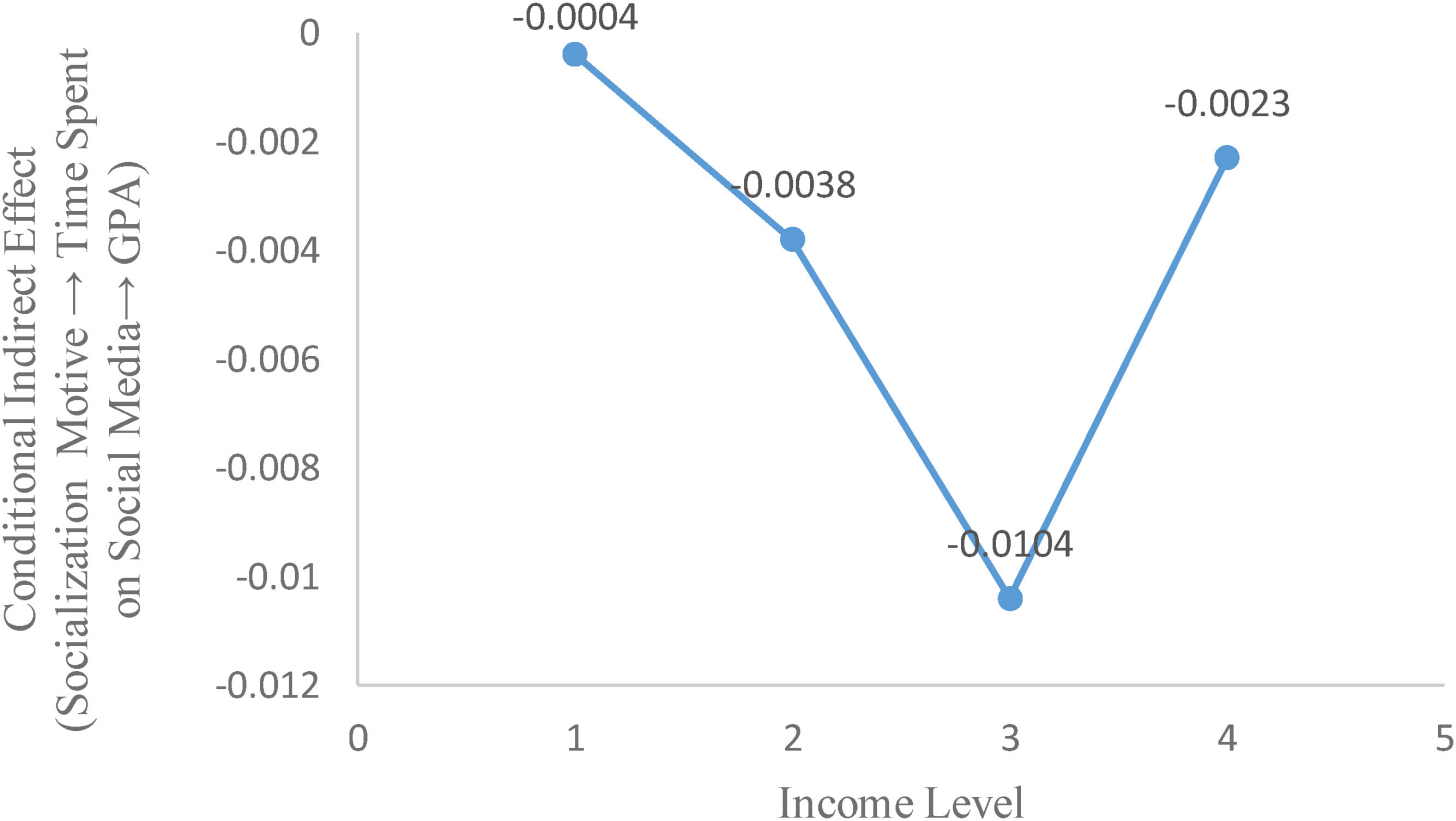
Conditional indirect effect of socialization motives on GPA through time spent on social networking sites across levels of income.

**FIGURE 6:**
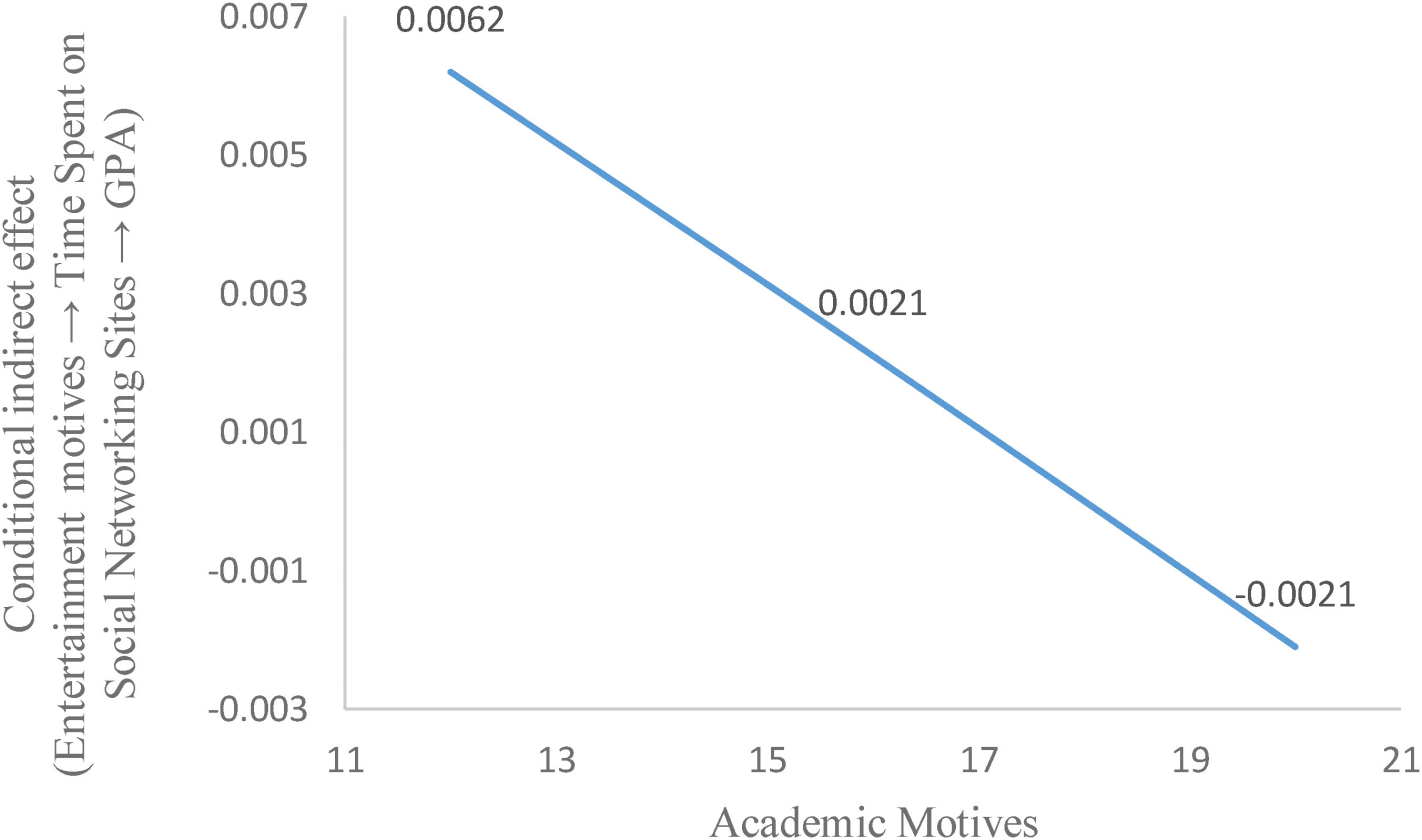
Conditional indirect effect of entertainment motivation on GPA through time spent on social networking sites levels of academic motives.

For brevity, Table 5 reports only statistically significant interaction effects predicting time spent on social networking sites, while Table 6 reports only statistically significant indices of moderated mediation. Conditional indirect effects by moderator level are summarized in Table 6 and illustrated in Figures 2–6. These estimates identify the moderator levels at which the indirect pathway was statistically significant and illustrate how the association between each motive and GPA varied across those levels. Moderated mediation was inferred when the bootstrap confidence interval for the index of moderated mediation did not include zero, consistent with PROCESS Model 7 decision criteria.

**TABLE 5:** Interaction effects predicting time spent on social networking sites (Path X → M; PROCESS model 7; *N* = 323).

Interaction term (X × W)	B	SE	*t*	*p*
Entertainment motives × academic motive	0.014	0.005	3.06	0.002
Socialization motives × entertainment motive	0.014	0.006	2.31	0.022
Socialization motive × academic year (year 3 vs. year 1)	–0.259	0.067	-3.86	< 0.001
Socialization motive × academic year (year 4 vs. year 1)	0.155	0.070	2.20	0.028
Socialization motive × no. of social networking sites accounts (6+ vs. 1)	–0.192	0.059	-3.24	0.001
Socialization motive × family income (>15,001 SAR vs. < 5,000 SAR)	–0.112	0.055	–2.05	0.041

*B*, unstandardized regression coefficient; *SE*, standard error. Multi-categorical moderators were dummy-coded with the lowest category serving as the reference group. Each interaction term represents a specific contrast relative to the reference category.

**TABLE 6:** Index of moderated mediation of social media use motives on GPA via time spent on social networking sites (PROCESS model 7; 5,000 bootstrap samples; *N* = 323).

Predictor (X)	Mediator (M)	Moderator (W)	Contrast (W)	Index	Boot *SE*	95% Bootstrap *CI*
Entertainment motives	Time spent on social networking sites	Academic motives	–	–0.0010	0.0004	[–0.0019, –0.0003]
Socialization motives	Time spent on social networking sites	Entertainment motives	–	–0.0010	0.0005	[–0.0021, –0.0002]
	Time spent on social networking sites	Academic year	Year 3 vs. Year 1	0.0180	0.0063	[0.0065, 0.0309]
	Time spent on social networking sites	No. of social networking sites accounts	6+ vs. 1	0.0126	0.0047	[0.0045, 0.0226]
	Time spent on social networking sites	Family income	Upper middle income vs. low income	0.0081	0.0043	[0.0003, 0.0173]

Indices are reported only when the 95% bias-corrected bootstrap confidence interval does not include zero. For multi-categorical moderators, indices represent pairwise contrasts relative to the reference category, consistent with PROCESS Model 7 dummy coding. Remaining social media use motives, sex, academic year, family income, and number of social networking sites accounts were included as controls.

### Conditional indirect effects—entertainment motives

As shown in Table 7, when academic motive was specified as the moderator, a significant conditional indirect effect was observed exclusively at low levels of academic motive [Effect = 0.006, 95% CI (0.0018, 0.0119)]. The indirect effects at moderate and high levels were not statistically significant, suggesting that the mediated relationship was confined to students with lower academic motivation for social media use.

**TABLE 7:** Conditional indirect effects of social media use motives on GPA via time spent on social networking sites (PROCESS model 7; *N* = 323; 5,000 bootstrap samples).

Predictor (X)	Moderator (W)	Moderator level	Effect	Boot *SE*	95% Bootstrap *CI*
Entertainment motives	Academic motives	Low	0.006	0.003	[0.0018, 0.0119]
		Medium	–0.002	0.002	[–0.0009, 0.0061]
		High	–0.002	0.002	[–0.0066, 0.0022]
Socialization motives	Entertainment motives	Low	0.001	0.003	[–0.0067, 0.0046]
		Medium	–0.005	0.002	[–0.0102, —0.0009]
		High	–0.009	0.003	[–0.0166, –0.0033]
	Family income	Low income	–0.000	0.007	[–0.0144, 0.0114]
		Lower-middle income	–0.004	0.003	[–0.0103, 0.0014]
		Upper-middle income	–0.010	0.004	[–0.0198, –0.0033]
		High income	–0.002	0.003	[–0.0089, 0.0032]
	Academic year	Year 1	–0.011	0.005	[–0.0228, –0.0025]
		Year 2	–0.009	0.003	[–0.0162, –0.0034]
		Year 3	0.009	0.005	[–0.0005, 0.0184]
		Year 4	–0.002	0.004	[–0.0104, 0.0041]
		Year 5	–0.003	0.003	[–0.0098, 0.0018]
	Number of SNS	1 Account	–0.004	0.012	[–0.0245, 0.0209]
		2 Accounts	–0.013	0.013	[–0.0367, 0.0108]
		3 Accounts	–0.007	0.004	[–0.0152, 0.0009]
		4 Accounts	–0.007	0.003	[–0.0131, –0.0015]
		5 Accounts	–0.009	0.004	[–0.0176, –0.0027]
		≥ 6 Accounts	0.003	0.003	[–0.0022, 0.0089]

Boot *SE*, bootstrap standard error. Confidence intervals are bias-corrected bootstrap intervals based on 5,000 samples. For continuous moderators, conditional indirect effects were estimated at the 16th, 50th, and 84th percentiles, consistent with Hayes’s recommendations. Categorical moderators are labeled using substantive category names rather than numeric codes.

### Conditional indirect effects—socialization motives

As shown in Table 7, conditional indirect effects for socialization motive differed across moderators. When entertainment motive served as the moderator, the conditional indirect effect of socialization motive was not statistically significant at low levels of entertainment motive but became significantly negative at moderate [Effect = –0.005, 95% CI (–0.0102, –0.0009)] and high levels [Effect = –0.009, 95% CI (–0.0166, –0.0033)]. This pattern indicates that the indirect pathway through time spent on social networking sites was observed at moderate and high levels of the moderator. When academic year served as the moderator, the indirect pathway through time spent on social networking sites was significantly negative in Year 1 [Effect = –0.011, 95% CI (–0.0228, –0.0025)] and Year 2 [Effect = –0.009, 95% CI (–0.0162, –0.0034)], whereas effects in subsequent years were not statistically significant, indicating that the mediated relationship was present primarily during the early stages of medical training. With family income as moderator, a significant negative indirect effect was observed only in the upper-middle income category [Effect = –0.010, 95% CI (–0.0198, –0.0033)], indicating that the indirect effect was limited to this income group.

Similarly, when the number of social media accounts was examined as the moderator, significant indirect effects were observed among students reporting four accounts [Effect = –0.0067, 95% CI (–0.0131, –0.0015)] and five accounts [Effect = –0.0092, 95% CI (–0.0176, –0.0027)], while effects at other account levels were not significant, indicating that the mediated association was most pronounced among students with a moderate number of accounts. Taken together, these findings demonstrate that the indirect effects of socialization motive on GPA through time spent on social networking sites were contingent on specific contextual and individual characteristics rather than uniformly present across all groups.

## Discussion

This study investigated how different motives for SNSs use relate to academic performance among medical students and whether these relationships are associated with the amount of time students spend on SNSs each day. Guided by U&G theory, SNS engagement was conceptualized as motive-driven rather than exposure-driven behavior. Separate pathways were therefore examined for academic, socialization, informational, and entertainment motives. In addition to assessing direct and mediated relationships, exploratory conditional process analyses were conducted to determine whether indirect effects varied across motivational and student characteristics. Taken together, the findings support a differentiated, association-based account of the relationship between SNS use and academic performance and extend recent motive–time–performance mediation models to the context of medical education.

### Direct associations

At the level of direct relationships, SNS motives did not show uniform relationships with either time spent on SNSs or GPA (Table 3). After adjusting for covariates, only socialization motives maintained a stable positive association with time spent, whereas other motives showed weaker or less consistent links with usage time. Overall, the magnitude of these effects was modest, indicating that motives explain only part of the variability in daily SNS engagement. This selective pattern is consistent with U&G theory, which proposes that media behaviors are shaped primarily by specific user needs and gratifications rather than exposure to the platform itself (Katz et al., 1974; Ruggiero, 2000; Quan-Haase and Young, 2010).

Across all models, daily time spent on SNSs demonstrated a consistent negative association with GPA (Table 3). Although this relationship was statistically reliable, the effect size was small to moderate, suggesting that SNS time is one of several factors related to academic performance rather than a dominant predictor. This finding aligns with previous studies linking heavier SNS use with lower academic performance (Junco, 2012; Jacobsen and Forste, 2011; Masalimova et al., 2023), yet the present results provide additional nuance by showing that time spent on SNSs is not merely a volume indicator—it is statistically associated with academic performance and may serve as an intervening variable linking motives and outcomes. In practice, time investment likely reflects not only duration but also attentional fragmentation, task switching, and reduced deep-work intervals, all of which are known to impair learning efficiency and academic performance (Junco, 2012; Karpinski et al., 2013; Feng et al., 2019). Thus, time can be interpreted as an associative bridge connecting motivational drivers and academic outcomes rather than serving solely as a descriptive usage metric.

Direct motive–GPA associations were limited but theoretically informative. Informational motives retained a negative direct association with GPA. One possible interpretation is that information-oriented SNS use may expose students to large volumes of diverse and rapidly changing content that could make it more difficult to focus on academically relevant information. Previous research has proposed mechanisms such as information overload and attentional fragmentation as potential explanations for such outcomes (Giunchiglia et al., 2018; Zavala et al., 2023). However, because these mechanisms were not directly assessed in the present study, they should be regarded as theoretical possibilities rather than explanations supported by the current data. Future research should directly examine these processes to clarify the mechanisms linking informational SNS use with academic performance. In contrast, academic motives showed a small positive overall association with GPA, consistent with evidence that academically directed SNS use tends to be more goal-bounded and strategically regulated (Chang et al., 2019; Ashraf et al., 2021). This contrast highlights that the underlying motive structure for SNS engagement influences not just why students log in, but also how cognitively organized their engagement tends to be.

### Indirect effects

The primary mechanism tested in this study was whether time spent on SNSs statistically mediates (i.e., is indirectly associated with) the relationship between motives and GPA (Tables 3, 4). The mediation results indicate that time spent is associated with these relationships as a selective intermediary rather than a universal mediator. Socialization motives demonstrated a statistically significant indirect effect on GPA through time spent in both unadjusted and covariate-adjusted models. However, the size of this indirect effect was modest, and its interpretation should be considered within the broader pattern of findings rather than in isolation. In contrast, after covariate adjustment, indirect effects for entertainment and informational motives were no longer statistically significant, and academic motives did not demonstrate any indirect effect. These results suggest variability in the robustness of mediation effects, highlighting that not all theoretically expected pathways were supported in the data.

This pattern suggests that socially driven SNS use is particularly likely to be associated with increased time exposure, which in turn linked to lower academic performance. Importantly, this interpretation is limited to socialization motives, as no comparable indirect effects were observed for academic, entertainment, or informational motives after adjustment. Previous research has shown that socially oriented SNS use is associated with more frequent checking and longer session duration (Al-Menayes, 2015; Quan-Haase and Young, 2010). Nevertheless, educational technology research indicates that social media can support learning when used for structured academic collaboration and peer support (Manca and Ranieri, 2016). The present findings suggest that, in this sample, socialization use was predominantly time-expansive rather than academically structured.

The mediation pattern observed in this study closely parallels recent findings reported by Cuong et al. (2025), who demonstrated that daily SNS time mediates the relationship between SNS use motives and GPA. In that study, social and communication motives produced negative indirect pathways through usage time, whereas academic motives displayed more protective patterns. The present findings partially align with this pattern, with evidence supporting mediation only for socialization motives, while other motive-based indirect pathways were not supported after covariate adjustment.

The time-based mediation pathway is also consistent with time-displacement and attentional competition explanations, which propose that SNS engagement competes with sustained study time and deep cognitive focus (Junco, 2012; Karpinski et al., 2013). From a U&G perspective, motives are associated with academic outcomes indirectly through behavioral exposure patterns, specifically the duration and intensity of engagement (Katz et al., 1974; Ruggiero, 2000).

### Conditional indirect effects

A major extension of the present findings comes from the exploratory moderated mediation analyses, which tested whether motive → time → GPA pathways vary in their statistical associations across motivational combinations and student characteristics (Figures 2–6 and Tables 5–7). Conditional process models recognize that mediation mechanisms may not operate uniformly across individuals and are conceptually consistent with U&G perspectives that emphasize the role of motive configurations and user context (Ruggiero, 2000; Hayes, 2018).

The most prominent conditional pattern showed that entertainment motives moderated the indirect effect of socialization motives through time spent. The interaction significantly predicted time spent (Table 5), and the index of moderated mediation was statistically significant (Table 6). Conditional indirect effects indicated that the socialization → time → GPA pathway was weak at low entertainment motive levels but became significantly negative at moderate and high levels (Table 7 and Figure 2). This pattern suggests a dual-gratification amplification effect in which relational and hedonic motives jointly intensify engagement duration. Motive research indicates that social media gratifications frequently co-occur and reinforce one another, producing compounded engagement patterns rather than isolated effects (Quan-Haase and Young, 2010; Al-Menayes, 2015).

Additional conditional effects were observed across academic year, family income, and number of SNS accounts (Figures 3–5 and Tables 6, 7). The time-mediated pathway for socialization motives was strongest among students in earlier academic years, particularly years 1 and 2, and attenuated in later years. This is consistent with research suggesting that early stage medical students experience greater adjustment demands and less stable self-regulatory learning patterns, making their study time more vulnerable to digital distraction (Avci et al., 2015; Findyartini et al., 2024). Similarly, conditional effects concentrated in specific account-breadth groups are also consistent with evidence that multi-platform use is associated with higher engagement frequency and attentional fragmentation (Junco, 2012; Karpinski et al., 2013).

A complementary conditional pattern indicated that academic motives moderated the entertainment → time → GPA pathway, such that the indirect effect of entertainment motives through time was present primarily at low levels of academic motive (Figure 6 and Tables 5–7). This suggests that academically oriented SNS use may function as a behavioral buffer that limits hedonic time expansion, consistent with prior evidence that task-related SNS activity is associated with more productive engagement and favorable academic outcomes than purely recreational use (Chang et al., 2019; Ashraf et al., 2021).

Because these moderated mediation analyses were specified as exploratory rather than preregistered tests, the identified boundary conditions should be interpreted as hypothesis-generating rather than confirmatory subgroup effects (Hayes, 2018). Accordingly, these findings should be interpreted with caution and viewed as preliminary patterns requiring replication in future research. Nevertheless, they add important nuance by demonstrating that motive → time → GPA pathways are not population-uniform but depend on motivational combinations and student context.

Although the regression models were statistically significant, their explanatory power was modest. The mediation model accounted for approximately 8% of the variance in daily SNS usage duration, while the final model explained approximately 11.5% of the variance in GPA. Consequently, a substantial proportion of the variability in academic performance remained unexplained by the variables included in the present study. These findings indicate that SNS motives and daily SNS use represent only one component of a much broader set of influences on academic achievement. Academic outcomes are shaped by numerous additional factors, including prior academic ability, learning strategies, sleep quality, mental health, study habits, self-regulated learning, attendance, and broader socioeconomic and institutional conditions. These influences are well documented in the literature (Zimmerman, 2002; Poropat, 2009; Credé and Kuncel, 2008). Therefore, the findings should be interpreted as supporting a specific, theory-driven pathway linking SNS motives, SNS use, and academic performance rather than as a comprehensive explanation of academic achievement.

### Novel contribution

This study advances SNS–academic performance research by integrating motive differentiation, behavioral mediation, and conditional process modeling within a single analytical framework. In doing so, it responds to recent calls for mechanism-oriented and motive-sensitive models of social media use in academic contexts (Masalimova et al., 2023) and directly addresses our objective of testing motive-specific, time-mediated, and conditional pathways linking SNS use with academic performance. By replicating and extending recent motive–time–GPA mediation findings (Cuong et al., 2025) within a medical education setting, the study offers a more precise explanation of how and under what conditions SNS use motives are associated with academically relevant outcomes.

### Practical implications for medical education

The findings suggest that educational strategies addressing SNS use should move beyond blanket time-reduction advice and instead focus on motive-sensitive and behaviorally targeted guidance. Because motives differ in how they translate into time investment and academic association, screening students only on total SNS time may overlook higher-risk motivational profiles. Students whose SNS use is primarily socially driven, particularly when combined with strong entertainment motives, appear more likely to develop time-expansive usage patterns that are academically consequential.

Medical education programs may therefore benefit from incorporating digital self-regulation and motive-awareness training into study-skills and professionalism curricula. Helping students reflect on why they use SNSs, how motives influence session duration, and how to apply time-bounding strategies may improve self-management. The conditional findings indicating stronger early year vulnerability suggest that first-year orientation and transition programs are especially appropriate targets for such interventions. Importantly, the results do not support a uniformly negative view of SNS use: academically oriented use showed no harmful time-based pathway and modest positive total associations with GPA, supporting approaches that encourage purposeful, academically aligned SNS engagement rather than blanket restriction.

## Conclusion

This study provides a motive-sensitive and association-based account of how SNS use relates to academic performance among medical students. Grounded in Uses and Gratifications theory, SNS use was conceptualized as purpose-driven behavior rather than uniform exposure, distinguishing academic, socialization, informational, and entertainment motives. The findings indicate that academic outcomes are shaped not simply by SNS use itself, but by the motives underlying use and how those motives are associated with differences in time investment. Across models, daily time spent on SNSs emerged as a consistent negative correlate of GPA, serving as an indirect statistical pathway through which specific motives—particularly socialization—were linked with poorer academic performance. Socialization motives showed a statistically significant indirect effect on GPA via increased SNS time, whereas academic, entertainment, and informational motives did not demonstrate reliable indirect effects after covariate adjustment. Academic motives exhibited no harmful time-based pathway and were modestly positively associated with performance. Informational motives retained a small negative direct association with GPA. However, the mechanisms underlying this association were not examined in the present study and therefore require further empirical investigation. Conditional process analyses further demonstrated that these pathways are not uniform across students: the indirect effect of socialization motives was amplified when entertainment motives were high and attenuated among students with stronger academic motives. Additional moderation by academic year and platform breadth indicates heightened vulnerability among early stage students and those with broader SNS ecosystems. Together, these findings extend recent motive–time–performance mediation research by replicating key mechanisms within a single-institution medical student sample, demonstrating that SNS-related academic risk is both motive-dependent and context-sensitive. The results support educational approaches that emphasize motive awareness, purposeful SNS engagement, and digital self-regulation rather than blanket restrictions on social media use. However, caution is warranted in generalizing these findings beyond the present institutional context, and future multi-institutional studies are needed to confirm their external validity.

### Limitations and future directions

Several limitations should be considered when interpreting these findings. First, the cross-sectional design precludes causal inference, and reciprocal relationships between SNS use and academic performance cannot be ruled out. Second, core variables, including time spent on SNSs and GPA, were self-reported and may be subject to reporting bias. Future studies should incorporate objective platform logs, device-based usage metrics, and verified academic records. GPA was measured using categorical ranges and approximated using midpoint values, which may have reduced precision compared to exact scores. However, supplementary analyses using ordinal regression produced a similar pattern of findings, suggesting that the results were not substantially affected by this operationalization. In addition, the current analysis focused on overall SNS time and did not differentiate between platform-specific or activity-specific engagement. Future research should distinguish passive browsing, interactive engagement, and academically oriented SNS behaviors. Although procedural and statistical steps were taken to reduce common method bias and multiple covariates were included, shared-method variance cannot be fully excluded. Moreover, reliance on Harman’s one-factor test represents a limited diagnostic approach and does not definitively rule out common method bias. Given that all key variables were self-reported, future research should employ more robust techniques, such as marker-variable approaches or confirmatory factor analysis (CFA) with a latent method factor, to better assess and control for potential common method variance. Third, although the Social Networking Usage Questionnaire (SNUQ) has been used in diverse cultural contexts and additional discriminant validity analyses in the present study supported the distinctiveness of its four dimensions, the study was not designed as a comprehensive scale-validation investigation. Consequently, the four-factor structure was not subjected to a full confirmatory measurement evaluation within the present sample. Future research should employ confirmatory factor analysis (CFA) and other structural validation procedures to further examine the stability and generalizability of the SNUQ factor structure across different cultural and educational contexts, particularly when the subdimensions are interpreted as motive-related forms of SNS engagement. Fourth, the study employed a convenience sampling strategy from a single institution (King Faisal University), which limits the generalizability of the findings to other medical schools or broader populations of medical students. In particular, students from one university may not fully represent the diversity of medical students across Saudi Arabia or other academic disciplines. Additionally, recruitment through WhatsApp groups may have introduced selection bias, as students who are more digitally active or frequently engaged with online communication platforms were more likely to be reached and to participate. This may have resulted in an overrepresentation of individuals with higher levels of social media engagement, which is directly relevant to the study variables. Consequently, estimates of SNS use and the magnitude of the observed associations among SNS motives, daily SNS usage duration, and GPA may not fully reflect those that would be observed in a more representative sample of medical students. Furthermore, because no data were collected from non-participants, it was not possible to formally assess whether participants differed systematically from those who declined participation. As a result, self-selection bias cannot be excluded. Therefore, the results should be interpreted within the specific context of the study setting, and caution is warranted when extending the findings to other institutions or cultural contexts. Future multi-institutional studies are needed to enhance external validity and confirm the robustness of the observed relationships across diverse academic environments. Finally, the moderated mediation analyses were exploratory and should be interpreted as hypothesis-generating rather than confirmatory. Given the number of interaction effects tested, there is an increased risk of Type I error, and statistically significant findings should be interpreted with caution. Future studies should apply appropriate corrections for multiple testing (e.g., Bonferroni or false discovery rate adjustments) and ideally preregister hypotheses to strengthen the robustness of conditional process findings. These effects should be preregistered and replicated in independent samples. Future research would benefit from longitudinal and multi-institutional designs to test causal pathways and from examining additional mediators such as procrastination, attentional fragmentation, and self-regulated learning. Such work would further clarify the mechanisms through which SNS motives shape academic outcomes and inform more targeted interventions in medical education.

## Data Availability

The raw data supporting the conclusions of this article will be made available by the authors, without undue reservation.
